# Can mindfulness play a role in building social-emotional capacities among youth exposed to screens?

**DOI:** 10.3389/fpsyt.2023.1165217

**Published:** 2023-06-22

**Authors:** Soyeon Kim, Stephanie Munten, Shavon Stafford, Nathan J. Kolla

**Affiliations:** ^1^Waypoint Centre for Mental Health Care, Waypoint Research Institute, Penetanguishene, ON, Canada; ^2^Psychiatry and Behavioural Neurosciences, McMaster University, Hamilton, ON, Canada; ^3^Centre for Addiction and Mental Health, University of Toronto, Toronto, ON, Canada

**Keywords:** mindfulness, self-compassion, resilience, self-esteem, youth, COVID-19

## Abstract

**Introduction:**

Increased screen time coupled with public safety restrictions may pose a serious challenge to adequate social-emotional development in youth during the pandemic. Social-emotional competence (resilience, self-esteem, and self-compassion) are essential for youth to adapt to the “new normal” in the prolonged pandemic timeline. The current study investigated the efficacy of a mindfulness-based intervention on youth social-emotional capacity while accounting for screen time.

**Methods:**

One hundred and seventeen youth participated in a 12-week, online mindfulness-based program and completed pre-, post- and follow-up surveys across five cohorts during the COVID-19 pandemic (spring 2021 to spring 2022). Changes in youths’ resilience (RS), self-esteem (SE), and self-compassion (SC) between the three-time points were examined using linear regression analyses (unadjusted, partially adjusted for screen time, and fully adjusted for demographic and screen time variables). The regression models accounted for demographic (age, sex), baseline mental health status, and screen time (passive, social media, video games, and educational types of screen-based behaviours) factors.

**Results:**

In an unadjusted regression model, resilience [*β* = 3.68, 95%CI = 1.78–5.50, *p* < 0.001], self-compassion [*β* = 0.50, 95%CI = 0.34–0.66, *p* < 0.001], and self-esteem [*β* = 2.16, 95%CI = 0.98–3.34, *p* < 0.001] significantly increased after the mindfulness program, and the effects were maintained in the follow-up. The efficacy of the mindfulness program persisted after controlling for five types of screen time [RS: *β* = 2.73, 95%CI = 0.89–4.57, *p* < 0.01; SC: *β* = 0.50, 95%CI = 0.32–0.67, *p* < 0.001; SE: *β* = 1.46, 95%CI = 0.34–2.59, *p* < 0.01] and in a fully adjusted model which additionally accounted for the baseline mental health status and demographic factors [RS: *β* = 3.01, 95%CI = 1.20, *p* < 0.01; SC: *β* = 0.51, 95%CI = 0.33–0.68, *p* < 0.001; SE: *β* = 1.64, 95%CI = 0.51–2.77, *p* < 0.01] and maintained its impact in the follow-up.

**Discussion:**

Our findings reinforce the evidence base on the efficacy of mindfulness and support the use of online mindfulness programs in building social–emotional competencies (i.e., self-compassion, self-esteem, and resilience) among youth exposed to screens during the pandemic.

## 1. Introduction

Recent significant global events (e.g., the COVID-19 pandemic) have disrupted the predictable and safe environment that facilitates healthy social-emotional development in youth (1–3). Adolescence through early adulthood is a critical developmental period for biological, social, neurodevelopmental, and psychological changes (4). One persistent concern throughout the past decade, exacerbated during the pandemic, is the increase in screen-based behaviors (2, 3, 5). Increased screen time coupled with public safety restrictions that impede social interaction may pose a severe challenge to adequate social-emotional development in youth (6). In this prolonged pandemic timeline, equipping youth with social-emotional capacities, such as self-compassion, self-esteem and resilience, is needed more than ever as we adapt to the new normal. Therefore, studies that evaluate the efficacy of programs targeted at improving social-emotional competence and psychological wellbeing are warranted to provide evidence-based support for youth.

As technology has evolved, youth use technological devices to a greater degree to connect than ever before (7–9). For example, hours of TV viewing during adolescent leisure time decreased slightly but was more than offset by a sharp increase in leisure time computer use (10). Since 2010, there has been a rise in the usage of handheld electronic devices (i.e., smartphones), resulting in greater access to new media and increased risk of mental and physical health concerns than those who spend more time on non-screen activities (11, 12). Recently, the pandemic has further increased screen time for adolescents worldwide, as many schools switched to virtual classes or online curriculums, and social gathering restrictions resulted in reduced face-to-face interactions with peers (13–15). In Canada, sedentary behaviour guidelines for children and youth recommend engaging in no more than 2 h of screen time per day. However, a survey of Canadian parents revealed that adolescents’ time spent on screens increased to approximately 5.9 h per day compared to the pre-pandemic period (2.6 h) (15). Among the various screen time types, social media usage has risen significantly among adolescents and young adults (16).

Increased screen time among youth is associated with adverse mental health outcomes, including loneliness and feelings of social isolation (17), low self-esteem (18), decreased happiness and life satisfaction (19), and reduced mindfulness (9, 20, 21). Missing important social cues and reduced depth of connection (i.e., relationship quality, trust, and empathy) associated with decreased face-to-face contact may contribute to the adverse impact of screen time on mental health outcomes (8, 9). Interestingly, adolescents with higher levels of coping behaviors and social support engaged in less screen time (14). On the other hand, very little is known about screen time’s effect on youth’s social-emotional capacities. While the relationship between screen time and resilience and self-compassion is relatively unknown, existing literature indicates that lower self-esteem is often associated with increased screen-based behaviors (>3 h per day), which may be related to reduced participation in other non-screen-based organized activities, such sports and clubs (22–24). Given the impact of increased screen time on mental wellbeing, there is a heightened need for support for youth.

In the face of the increasing screen time trend during the pandemic and its adverse mental health impacts among youth, Polizzi et al. (25) highlight the importance of acceptance-based coping in recovery from COVID-19. The authors indicate that fully accepting emotion is critical to achieving longer-term resiliency. Resiliency, defined as “the ability to maintain relatively stable, healthy levels of psychological and physical functioning… as well as the capacity for generative experiences and positive emotions (pp. 20–21)” (26), may help youth to buffer adverse mental health effects from the pandemic. Besides resilience, adequate self-esteem, which refers to our feeling of worthiness as individuals, often contingent on successful attainment (27), may also support youth exposed to screens. Lastly, self-compassion, being caring and compassionate to oneself when faced with adverse events stemming from personal and external circumstances (27, 28), is associated with greater life satisfaction, emotional intelligence, social connectedness, less self-criticism, and thought suppression (29). A recent study also showed that coupled with increased resilience, self-compassion reduces anxiety related to challenging events, such as COVID-19 (30).

Mindfulness-based programs have become a popular way to improve overall wellbeing. Mindfulness-based interventions (MBIs) are a validated educational approach to building coping skills and resilience. With over two decades of mounting scientific research demonstrating benefits—from fortifying the immune system to reducing stress and anxiety and improving overall wellbeing—MBIs have been used to mitigate emotional challenges (31–33). Secular mindfulness is a practice that involves focusing awareness on the present moment while acknowledging and accepting one’s feelings, thoughts, and bodily sensations without judgment (34). More specifically, MBIs improve self-esteem, self-concept and social competencies, allowing participants to become more resilient (35, 36). Self-esteem has also partially mediated the relationship between mindfulness and wellbeing (37). Yet, studies that sampled young adolescents are relatively scarce, despite the critical importance of this age period in meeting social-emotional developmental milestones (38). Further, studies that examine MBIs’ impact on improvements in self-compassion remain limited (39) despite it being an important factor in wellbeing intervention (40).

Given the increasing trend of screen-based activities among youths and its adverse impact on mental health, protecting youth by providing support for building social-emotional competencies is imperative now more than ever. However, it is unknown whether mindfulness can play a role in building social-emotional competencies (i.e., self-compassion, self-esteem, and resilience) among youth exposed to increased screen time during the pandemic. Additionally, research on youth encompassing early adolescence to young adulthood is needed to confirm the developmental relevance of implementing mindfulness programs for this age group. Therefore, this study aimed to investigate the efficacy of a mindfulness-based intervention on social-emotional capacity (self-esteem, self-compassion and resilience) in youth accounting for their screen time. We hypothesized that participation in a mindfulness-based program would improve youth’s social-emotional capacity. Our study provides evidence that could lead to developing new policies, practices, and programs focused on enhancing social-emotional competence among youths exposed to excessive screen time in this prolonged pandemic timeline.

## 2. Methods

### 2.1. Procedures and participants

Community youth from central- and north-central Ontario, Canada, were recruited using digital flyers and word of mouth at five-time points during the COVID-19 pandemic (early spring 2021, late spring 2021, fall 2021, winter 2022, spring 2022), each recruited for up to 8-weeks. The final sample consisted of pre-, post-, and 30-day follow-up-survey data from the five cohorts. The average age was 16.8 years old (SD = 3.7; range 12–25), and 78.4% were females. Informed consent and survey responses were collected in the REDCap, a secure online data repository system. An informational letter was provided to the parents of participants 15 years of age and younger. After obtaining consent, a pre-survey (*n* = 117) was administered before the mindfulness intervention began. Each mindfulness session ran live once a week for 1h, with two certified Mindfulness Ambassador Program (MAP) facilitators assigned to each group (max 20). A post-survey (*n* = 53) was administered immediately following the conclusion of the 12-week intervention, with a follow-up survey (*n* = 47) 1 month after the last session. Post-surveys were sent to participants who attended at least one session of the mindfulness intervention. Upon completing each post and follow-up survey, participants were offered a $25 gift card as a thank-you gift for their time. The institution’s Research Ethics Board approved all components of this study (HPRA# 21.03.02).

### 2.2. Intervention

The mindfulness intervention utilized for this study was the Mindfulness Ambassador Program (MAP, developed by a non-profit organization, Mindfulness Without Borders) (41, 42). The MAP is a structured, 12-week, evidence-based group-mindfulness program. Participants were asked to attend weekly 1-h sessions, each having an intentional theme with embedded social and emotional capacity-building practices (i.e., inspirational quotes, dialog prompts, mindfulness practices, journaling) that promote group discussion and learning with opportunities for individual reflection. Fundamental to MAP’s curriculum is the practice of mindfulness skills to create space for authentic discussions that emphasize participation, self-reflection, critical thinking, and perspective-taking, ultimately enhancing social and emotional competence. In light of the COVID-19 pandemic, the MAP was retooled from a face-to-face program to a virtual one. All 12 MAP sessions were offered live online using Zoom and led by two MAP-certified facilitators with one observer and a maximum of 20 participants. To maintain the consistency of the program facilitation across the cohorts, a fidelity checklist was mandatory for facilitators to fill out after each session.

### 2.3. Measures

#### 2.3.1. Demographics

Demographic information, including age and sex assigned at birth, was collected. Participant age and sex assigned at birth were dichotomized as “adolescent” (12–17 years) or “young adult” (≥18 years) and “male” or “female,” respectively.

#### 2.3.2. Baseline mental health

The Strengths and Difficulty Questionnaire (SDQ(S) 11–17), a scale designed to measure emotional and behavioral problems, such as hyperactivity-inattention, and peer problems in youth (43) was used to assess participants’ baseline mental health. The psychometric properties of the SDQ, such as internal consistency (Cronbach α: 0.73) and retest stability after 4 to 6 months (mean: 0.62), have been well established (44). The first 25 items consist of five scales (emotional symptoms, conduct problems, hyperactivity, peer problems, and prosocial) with five items each. Items are rated on a 3-point Likert scale ranging from 0 (Not true) to 2 (Certainly true), with five reverse-scored items. Scores are summed to compute the total difficulties score (range 0–40), where higher scores indicate greater problems. Total scores ranging from 0–15 were coded as “low risk for significant problems,” 16–19 as “moderate risk for the significant problem,” and 20–40 as “high risk for significant problems (45).”

#### 2.3.3. Screen time

Participants were asked to complete a questionnaire with four self-report items designed to estimate how much time (hours), on average, they spent engaging in various types of screen time per day over the past 7 days. Screen types were defined as Pleasure ([passively watching] TV, movies or videos), social media (Facebook, Instagram, Snapchat, etc.), Video Games ([playing] video games (online and/or offline)), and Education ([use of] an electronic device (i.e., computer, laptop, tablet) for educational purposes (i.e., schooling)). Approximate time spent on each item was measured on a 4-point Likert scale ranging from 1 to 4, and response options included: less than 1 h, 1–3 h, 3–5 h, and more than 5 h.

#### 2.3.4. Resilience

The Nicholson McBride Resilience Questionnaire [NMRQ; (46)] was used to measure participants’ resilience. The NMRQ is a self-report questionnaire designed to measure resilience, defined as an individual’s capacity to bounce back from extreme occasions or triumph in the face of hardship (46). The NMRQ consists of 12 Likert-type questions scored on a scale of 1 (*strongly disagree*) to 5 (*strongly agree*), where higher scores are indicative of greater resilience. Scores are summed to compute the total score. Total scores ranging from 1 to 37 are classified as “developing,”; 38 to 43 as “established,”; 44 to 48 as “strong,”; and 49 to 60 as “exceptional.”

#### 2.3.5. Self-compassion

The Self-Compassion Scale [SCS; (28)] was used to measure participant’s self-compassion, which assesses six different aspects of self-compassion to create an overall self-compassion score: Self-Kindness (e.g., “I try to be understanding and patient toward aspects of my personality I do not like”), Self-Judgment (e.g., “I’m disapproving and judgmental about my own flaws and inadequacies”), Common Humanity (e.g., “I try to see my failings as part of the human condition”), isolation (e.g., “When I think about my inadequacies it tends to make me feel more separate and cut off from the rest of the world”), mindfulness (e.g., “When something painful happens I try to take a balanced view of the situation”), and Over-Identification (e.g., “When I’m feeling down I tend to obsess and fixate on everything that’s wrong”). These subscales are highly inter-correlated, and a single higher-order factor (i.e., self-compassion) explains their inter-correlations (28). The SCS is a self-report questionnaire for individuals aged 12 and up consisting of 26 items representing both compassionate and uncompassionate ways to respond to oneself when facing adversity (28). Items are rated on a 5-point Likert scale ranging from 1 (*Rarely*) to 5 (*Almost always*). Total self-compassion scores are calculated by reverse scoring the negative subscale items (self-judgment, isolation, and over-identification) and then computing a total mean. Mean scores range from 1 to 5, where higher scores are indicative of greater self-compassion.

#### 2.3.6. Self-esteem

The Rosenberg Self-Esteem Scale [RSES; (47)] was used to measure participants’ self-esteem. The RSES is a self-report measure designed to assess adolescent and adult global self-esteem, defined as the overall appraisal of one’s worth or value as a person (47, 48). The RSES includes 10 items, half representing generally positive feelings toward oneself (e.g., “On the whole, I am satisfied with myself”) and the other half representing generally negative feelings toward oneself (e.g., “At times, I think I am no good at all”). Items are rated on a 4-point Likert scale ranging from 0 (*Strongly Disagree*) to 3 (*Strongly Agree*), with the five items describing generally negative feelings toward oneself being reverse-scored. Scores are summed to compute a total score ranging from 0 to 30, where higher scores indicate greater self-esteem, and those between 15 and 25 are considered average. Scores ranging from 0–14 were coded as “low self-esteem,” 15–25 as “moderate self-esteem,” and 26–30 as “high self-esteem.”

### 2.4. Analysis

The efficacy of the MAP on adolescent resilience, self-esteem and self-compassion was examined at the three survey time points (pre-, post-, and follow-up) using linear regression analyses utilizing the Generalized Least Squares (GLS) Maximum Likelihood (ML), unstructured model. GLS is an extension of generalized linear models for panel (longitudinal) data that estimates more efficient and unbiased regression parameters (49). GLS adjusts the standard errors and produces efficient estimates of the coefficients by considering the over-time correlations when producing the coefficient estimates. We also applied ML estimation to handle missing data on the response variables and an unstructured approach to account for less than 5-time points and a relatively small sample size. A series of regression models were used to examine the efficacy of mindfulness intervention on social-emotional capacities. First, the unadjusted model was conducted to evaluate the change in resilience, self-esteem and self-compassion scores individually over time. Next, the four types of screen time (passive, social media, video games, educational) were added (partially adjusted model) to test if the the efficacy of mindfulness intervention in outcome variables remains the same after accounting for screen time. Finally, participant characteristics (sex and age) and baseline mental health status (total SDQ score at pre-survey) were added (fully adjusted model) to account for correlates. Additionally, the role of four types of screen time on outcome variables was tested with linear regression analysis using pre-survey data. Coefficients and 95% confidence intervals (CIs) data are reported. Software for Statistics and Data Science (STATA; V.16.0) was used.

## 3. Results

Table 1 describes the demographic distribution, mental wellbeing scores, and screen type and time sub-categories. Forty one percentage of youth had a high-risk baseline mental health status, indicating a substantial risk of clinically significant problems. The majority of youths spent the most time (≥5 h/day) engaging in education-related screen time and less time on video games (<3 h/day) across all time points. It is worth noting that less than 3 h of screen time on all types increased after the intervention. For example, 38.4% of youth engaged in passive screen time less than 3 h before the intervention, and it increased to 52.9% immediately after the intervention and 55.4% after 1 month following the program. Youth average resilience score was “developing” at baseline (34.7 ± 7.3) and was improved to “established” immediately after participation in the mindfulness program (38.7 ± 7.6) and at follow-up (40.0 ± 7.8). Similarly, the youth self-esteem average score was low at baseline (13.6 ± 5.9) but was improved immediately after the program (15.3 ± 6.9) and remained improved at follow-up 30 days later (17.1 ± 6.4). For all outcome variables (resilience, self-compassion, and self-esteem), young adults had higher mean scores compared to adolescents at all three-time points.

**Table 1:** Distribution of participant demographics at baseline, outcome variable mean scores and screen time at each time point.

	Pre-survey (*N* = 117)	Post-survey (*N* = 53)	Follow-up survey (*N* = 47)
**Mental health status**			
Low risk	31.3%		
Moderate risk	27.8%		
High risk	40.9%		
**Social-emotional capacities**			
Resilience	34.7 ± 7.3	38.7 ± 7.6	40.0 ± 7.8
Self-compassion	2.3 ± 0.6	2.7 ± 0.9	2.9 ± 0.9
Self-esteem	13.6 ± 5.9	15.3 ± 6.9	17.1 ± 6.4
**Passive screen time**			
<1 h/day	5.1%	3.8%	12.8%
1–3 h/day	33.3%	49.1%	42.6%
3–5 h/day	24.8%	28.2%	21.2%
>5 h/day	36.8%	18.9%	23.4%
**Social media**			
<1 h/day	23.1%	28.2%	31.9%
1–3 h/day	29.1%	32.1%	31.9%
3–5 h/day	17.9%	18.9%	23.4%
>5 h/day	29.9%	20.8%	12.8%
**Video games**			
<1 h/day	45.3%	47.2%	59.6%
1–3 h/day	30.8%	32.1%	23.4%
3–5 h/day	13.7%	7.5%	8.5%
>5 h/day	10.3%	13.2%	8.5%
**Educational screen time**			
<1 h/day	7.7%	7.5%	19.6%
1–3 h/day	15.3%	15.1%	23.9%
3–5 h/day	19.7%	17.0%	17.4%
>5 h/day	57.3%	60.4%	39.1%

In an unadjusted model (Table 2), compared to the baseline, post-time point significantly associated with increased resilience [*β* = 3.65, 95%CI = 1.78–5.50, *p* < 0.001], self-compassion [*β* = 0.50, 95%CI = 0.34–0.66, *p* < 0.001], and self-esteem [*β* = 2.16, 95%CI = 0.98–3.34, *p* < 0.001]. These positive and significant associations maintained at follow-up for resilience [*β* = 4.51, 95%CI = 2.53–6.49, *p* < 0.001], self-compassion [*β* = 0.51, 95%CI = 0.34–0.67, *p* < 0.001] and self-esteem [*β* = 3.18, 95%CI = 1.94–4.41, *p* < 0.001]. Before accounting for screen time in the model (partially adjusted model; Table 2), we examined the relationship between four types of screen time on each outcome variable (i.e., resiliency, self-compassion, and self-esteem). Table 3 shows that more than 5 h of a passive screen time is significantly associated with decreased resilience [*β* = −6.89, 95%CI = −13.30 to −0.49, *p* = 0.035].

**Table 2:** Associations between outcome measure (resilience, self-compassion, self-esteem) and screen time and participant demographics (coefficients, 95% confidence intervals).

	Resilience			Self-compassion			Self-esteem		
	Unadjusted model	Partially adjusted model	Adjusted model	Unadjusted model	Partially adjusted model	Adjusted model	Unadjusted model	Partially adjusted model	Adjusted model
**Time**									
Pre	Ref	Ref	Ref	Ref	Ref	Ref	Ref	Ref	Ref
Post	3.6 5 (1.78–5.50)***	2.73 (0.89–4.57)**	3.01 (1.20–4.82)**	0.50 (0.34–0.66)***	0.50 (0.32–0.67)***	0.51 (0.33–0.68)***	2.16 (0.98–3.34)***	1.46 (0.34–2.59)*	1.64 (0.51–2.77)**
Follow up	4.51 (2.53–6.49)***	2.73 (0.71–4.74)**	3.10 (1.12–5.09)**	0.51 (0.34–0.67)***	0.46 (0.28–0.64)***	0.48 (0.31–0.66)***	3.18 (1.94–4.41)***	1.96 (0.63–3.29)**	2.37 (1.07–3.68)***
**Baseline mental health status**									
Low risk			Ref			Ref			Ref
Moderate risk			−3.04 (−5.90 to −0.17)*			−0.36 (−0.64 to −0.07)*			−2.29 (−4.64 to 0.06)
High risk			−5.61 (−8.32 to −2.90)***			−0.67 (−0.93 to −0.40)***			−5.74 (−7.94 to −3.54)***
**Sex**									
Female			Ref			Ref			Ref
Male			1.95 (−0.98 to 4.87)			−0.01 (−0.30 to 0.28)			0.79 (−1.59 to 3.16)
**Age**									
Adolescent			Ref			Ref			Ref
Young Adult			0.30 (−0.02 to 0.61)			0.05 (0.02 to 0.08)**			0.34 (0.09 to 0.60)**
**Passive screen time**									
<1 h/day		Ref	Ref		Ref	Ref		Ref	Ref
1–3 h/day		−2.73 (−5.99 to 0.52)	−2.39 (−5.64 to 0.86)		−0.13 (−0.37 to 0.12)	−0.14 (−0.39 to 0.11)		−0.89 (−3.13 to 1.35)	−0.86 (−3.07 to 1.36)
3–5 h/day		−3.15 (−6.27 to −0.03)*	−2.69 (−5.81 to 0.43)		−0.06 (−0.28 to 0.16)	−0.07 (−0.30 to 0.16)		−0.82 (−3.07 to 1.43)	−0.66 (−2.91 to 1.55)
>5 h/day		−6.30 (−9.74 to −2.86)***	−5.37 (−8.83 to −1.92)**		−0.28 (−0.54 to −0.01)*	−0.24 (−0.51 to 0.03)		−3.82 (−6.27 to −1.36)**	−3.37 (−5.78 to −0.96)**
**Social media**									
<1 h/day		Ref	Ref		Ref	Ref		Ref	Ref
1–3 h/day		−1.20 (−3.58 to 1.17)	−1.50 (−3.84 to 0.52)		0.15 (−0.06 to 0.12)	0.07 (−0.13 to 0.27)		−0.001 (−1.76 to 1.76)	−0.15 (−1.86 to 1.56)
3–5 h/day		0.34 (−2.31 to 2.99)	−0.03 (−2.71 to 2.64)		0.20 (−0.02 to 0.42)	0.12 (−0.11 to 0.34)		0.99 (−1.01 to 3.00)	0.66 (−1.33 to 2.66)
>5 h/day		−1.28 (−4.04 to 1.49)	−1.37 (−4.19 to 1.44)		0.17 (−0.05 to 0.40)	0.08 (−0.16 to 0.31)		−0.71 (−2.91 to 1.50)	−0.89 (−3.06 to 1.29)
**Video game**									
<1 h/day		Ref	Ref		Ref	Ref		Ref	Ref
1–3 h/day		−1.14 (−3.19 to 0.90)	−1.54 (−3.59 to 0.52)		0.06 (−0.10 to 0.22)	0.07 (−0.09 to 0.24)		−0.07 (−1.68 to 1.55)	0.07 (−1.48 to 1.62)
3–5 h/day		−2.01 (−4.86 to 0.85)	−1.52 (−4.42 to 1.38)		−0.03 (−0.26 to 0.19)	0.06 (−0.17 to 0.29)		−1.12 (−3.17 to 0.93)	−0.29 (−2.32 to 1.75)
>5 h/day		−3.50 (−6.92 to −0.08)*	−3.00 (−6.37 to 0.37)		−0.12 (−0.39 to 0.15)	−0.05 (−0.31 to 0.22)		−2.59 (−4.98 to −0.20)*	−1.44 (−3.77 to 0.88)
**Educational screen time**									
<1 h/day		Ref	Ref		Ref	Ref		Ref	Ref
1–3 h/day		0.15 (−2.80 to 3.09)	0.13 (−2.76 to 3.02)		0.04 (−0.17 to 0.25)	0.07 (−0.15 to 0.28)		2.53 (0.35 to 4.72)	2.39 (0.27 to 4.52)
3–5 h/day		−2.38 (−5.59 to 0.82)	−2.21 (−5.35 to 1.39)		−0.02 (−0.26 to 0.21)	−0.02 (−0.26 to 0.22)		1.61 (−0.71 to 3.92)	1.41 (−0.83 to 3.66)
>5 h/day		−1.53 (−4.24 to 1.18)	−1.08 (−4.16 to 0.92)		−0.10 (−0.30 to 0.10)	−0.04 (−0.24 to 0.17)		1.12 (−0.87 to 3.10)	1.21 (−0.73 to 3.14)

*p < 0.05, **p < 0.01, ***p < 0.001.

**Table 3:** Associations between outcome measure (resilience, self-compassion, self-esteem) and screen time (coefficients, 95% confidence intervals) at baseline (pre-survey).

	Resilience	Self-compassion	Self-esteem
**Passive screen time**			
<1 h/day	Ref	Ref	Ref
1–3 h/day	−3.88 (−10.10 to 2.65)	5.02 (−6.48 to 16.52)	−0.29 (−0.89 to 0.31)
3–5 h/day	−2.71 (−9.32 to 3.90)	−0.09 (−11.79 to 11.62)	−0.26 (−0.86 to 0.36)
>5 h/day	−6.89 (−13.30 to −0.49)*	−0.15 (−11.45 to 11.16)	−0.55 (−1.14 to 0.04)
**Social media**			
<1 h/day	Ref	Ref	Ref
1–3 h/day	−0.29 (−4.16 to 3.58)	1.74 (−5.11 to 8.59)	0.14 (−0.22 to 0.50)
3–5 h/day	1.67 (−2.88 to 6.22)	0.85 (−7.11 to 8.81)	0.18 (−0.25 to 0.60)
>5 h/day	0.18 (−3.96 to 4.31)	−3.97 (−11.24 to 3.30)	0.18 (−0.21 to 0.56)
**Video game**			
<1 h/day	Ref	Ref	Ref
1–3 h/day	0.14 (−3.15 to 3.43)	−1.85 (−7.67 to 3.96)	0.05 (−0.26 to 0.35)
3–5 h/day	−2.29 (−6.66 to 2.08)	5.64 (−1.93 to 13.21)	0.04 (−0.36 to 0.43)
>5 h/day	−2.09 (−7.12 to 2.94)	−1.29 (−10.65 to 8.08)	−0.13 (−0.61 to 0.34)
**Educational screen time**			
<1 h/day	Ref	Ref	Ref
1–3 h/day	0.59 (−5.51 to 6.68)	−1.08 (−11.94 to 9.77)	0.16 (−0.41 to 0.72)
3–5 h/day	−4.04 (−9.87 to 1.79)	3.32 (−6.91 to 13.55)	0.03 (−0.51 to 0.56)
>5 h/day	−2.36 (−7.77 to 3.05)	2.50 (−6.99 to 11.98)	0.01 (−0.49 to 0.51)

*p < 0.05.

When adjusted for four screen time types (partially adjusted model; Table 2), the associations between three-time points and all outcome measures (resilience, self-compassion and self-esteem) remained unchanged. Passive screen time (>5 h/day) had a significant association with reduced resilience [*β* = −6.30, 95%CI = −9.74 to −2.86, *p* < 0.001], self-compassion [*β* = −0.28, 95%CI = −0.54 to −0.01, *p* = 0.042], and self-esteem [*β* = −3.82, 95%CI = −6.27 to −1.36, *p* = 0.002]. Further, video games (>5 h/day) also had a significant association with reduced resilience [β = −3.50, 95%CI = −6.92 to −0.08, *p* = 0.045] and self-esteem [β = −2.59, 95%CI = −4.98 to −0.20, *p* = 0.034], but not significantly associated with self-compassion.

Finally, the associations between the three-time points and resilience, self-compassion and self-esteem remained significant after adjusting for baseline mental health status (SDQ) and demographic factors (sex, age), in addition to screen time (fully adjusted model; Table 2). Moderate risk mental health status was associated with reduced resilience [*β* = −3.04, 95%CI = −5.90 to −0.17, *p* = 0.038] and self-compassion [*β* = −0.36, 95%CI = −0.64 to −0.07, *p* = 0.013], while high-risk mental health status was negatively associated with all three outcome measures (resilience [*β* = −5.61, 95%CI = −8.32 to −2.90, *p* < 0.001], self-compassion [*β* = −0.67, 95%CI = −0.93 to −0.40, *p* < 0.001], and self-esteem [*β* = −5.74, 95%CI = −7.94 to −3.54, *p* < 0.001]). Older age was positively associated with self-compassion [*β* = 0.05, 95%CI = 0.02–0.08, *p* = 0.002] and self-esteem [*β* = 0.34, 95%CI = 0.09–0.60, *p* = 0.007]. Adjusting for baseline mental health status, age and sex, passive screen time (>5 h/day) remained significantly negatively associated with resilience [*β* = −5.37, 95%CI = −8.83 to −1.92, *p* = 0.002], and self-esteem [*β* = −3.37, 95%CI = −5.78 to −0.96, *p* = 0.006]. Additionally, as the passive type of screen time was significantly associated with reduced resilience (Table 3), an interaction term (time by passive screen time) was added to the fully adjusted model to probe whether the effect of mindfulness intervention over time on resilience was modified by the amount of time spent on passive screen time. The result suggests that passive screen time up to 5 h per day does not influence the positive efficacy of mindfulness intervention [1–3 h of passive screen time at post survey: *β* = 9.41, 95%CI = 0.72–18.10, *p* < 0.05; 3–5 h of passive screen time at post survey: *β* = 9.60, 95%CI = 0.86–18.33, *p* < 0.05]. However, MBI was not significantly associated with enhanced resilience for those who engaged with 5 or more hours of passive screen (Table A1).

## 4. Discussion

Providing youth with evidence-based support for building social-emotional capacities is much needed, given the increasing trend of screen-based activities and their adverse impact on mental health. This study examined the efficacy of a mindfulness-based intervention on self-esteem, self-compassion and resilience in youth exposed to screen time during the pandemic. Our findings suggest a positive and significant role of a mindfulness-based intervention in enhancing social-emotional capacities among youth who are regularly exposed to different types of screen time (passive, social media, video games, and educational). It is worth noting that the change in resilience, self-compassion, and self-esteem after the mindfulness program has been maintained after 30 days of the program completion.

Our findings align with previous literature on the beneficial impact of MBIs on emotional challenges (31–33), resilience (35, 36), self-esteem (37), and self-compassion (39). Further, our findings uniquely add to the literature for accounting for the four types of screen time in the analysis adapting the current trend of increased screen time among youth. The magnitude of change in resilience, self-compassion, and self-esteem over time remained consistent after accounting for baseline mental health status, age, sex, and four types of screen time. We also included young adolescents (>12 years old) and examined MBI’s impact on self-compassion, which is scarce in the current literature (38, 39).

In this prolonged pandemic timeline, supporting youth to be resilient and to build social-emotional capacities is critical as our society recovers from a global mental health crisis. Our findings add to the growing literature on the efficacy of mindfulness practice on social-emotional capacity during the pandemic (50–52). Mindfulness programs’ common element embodying awareness and acceptance-based practices promoting connectedness may have contributed to building social-emotional capacity during crisis, such as the COVID-19 pandemic (25, 51). Also, mindfulness practices to awaken present-moment awareness and practice non-judgmental awareness can lead to positive mental health outcomes during the pandemic, such as relieving pandemic-related distress and emotional problems such as anxiety and depression during the pandemic (50, 52–54). For example, our findings indicate that 41% of youth had a high-risk baseline mental health status, which suggests that some youth may have met the threshold for clinical internalizing and externalizing disorders. Internal psychological factors such as self-evaluation (e.g., self-esteem) and coping capacity (e.g., resilience) that were significantly associated with the mindfulness intervention in the current study are suggested to mitigate anxiety and depression symptoms in youth (30, 55, 56). Therefore, our findings may indicate that online mindfulness programs may help prevent the development of depression and anxiety symptoms among community youth exposed to screen time during the pandemic and beyond.

Acknowledging the increasing trend of screen time among youth, we also examined the relationship between four types of screen time (passive, social media, video game and educational) and social-emotional capacity (resilience, self-compassion, and self-esteem) among youth during the COVID-19 pandemic. Our findings uniquely contribute to the literature as these associations are vastly understudied, particularly during the pandemic. Our findings suggest that extensive passive screen time (>5 h/day) is significantly associated with reduced resilience, accounting for other types of screen time. Further, MBI was not significantly associated with enhanced resilience for those who engaged with 5 or more hours of passive screen. Bouncing back from challenging situations is a critical capacity required to navigate the unprecedented pandemic era (25, 57). Given this evidence, parents, caregivers and educators should strategize promoting healthy screen hygiene in youth, such as encouraging screen-free time and moderating passive type of screen time through educational, public health and health promotion campaigns (58).

There are several limitations to our study. First, youth who participated in the study are primarily located in rural Ontario province and mostly females (78.4%), limiting the ability to generalize the findings. Future studies should consider recruiting youth with a balanced sex ratio and from diverse regions to ensure the results are replicable in a broader youth population. Second, the data collection was conducted on five-time points from 2021 to 2022 as we implemented the mindfulness programs in small groups. Due to this data collection approach, it is hard to examine the impact of a specific period during the pandemic (e.g., lockdown). Additionally, screen time was collected using a self-report measure where participants responded in categories (<1 h, 1–3 h, 3–5 h, >5 h), which only approximated the amount of time spent using screens for the week preceding each survey. This data collection approach lacks precision and granularity. Future studies should look to implement more robust logging of screen time to increase data accuracy. Lastly, almost half of the participants did not complete post and follow-up surveys resulting in a relatively low sample size. To adjust for the retention issue, we used linear regression models using the GLS, Maximum Likelihood (ML) approach that produces efficient/accurate estimates of the coefficients by considering the over-time correlations. ML estimation handles missing data on the response variables, and the unstructured approach accounts for less than 5-time points and a relatively small sample size (59). Future studies should incorporate a randomized controlled trial design to confirm current findings on the efficacy of mindfulness on youth social-emotional capacities.

Despite the limitations, the outcome of this study responds to the critical need to cultivate a resilient society amid a global pandemic. Lessons learned can also guide wellness programming focused on enhancing social-emotional competence among youths who are increasingly exposed to screens. The evidence base produced through this study will support the widespread implementation of an online mindfulness-based intervention delivered through physical distancing and beyond as we slowly transition to a “new normal.”

## Data availability statement

The raw data supporting the conclusions of this article will be made available by the authors, without undue reservation.

## Ethics statement

The studies involving human participants were reviewed and approved by Waypoint Research Ethics Board. Written informed consent to participate in this study was provided by the participants’ legal guardian/next of kin.

## Author contributions

SK conceived and designed the study. SK and SM managed the dataset and conducted the statistical analysis. SK, SM, SS, and NK discussed and interpreted the results. SK drafted the manuscript, and all authors provided critical reviews and contributed to the final manuscript.

## Funding

This study was supported by an Insight Development Grant funded by the Social Sciences and Humanities Research Council of Canada (Award # 430-2020-00288) and the Bell Community Fund Grant.

## Conflict of interest

The authors declare that the research was conducted in the absence of any commercial or financial relationships that could be construed as a potential conflict of interest.

## Publisher’s note

All claims expressed in this article are solely those of the authors and do not necessarily represent those of their affiliated organizations, or those of the publisher, the editors and the reviewers. Any product that may be evaluated in this article, or claim that may be made by its manufacturer, is not guaranteed or endorsed by the publisher.

## Figures and Tables

**Table A1:** Interaction between time and passive screen time on resilience (coefficients, 95% confidence intervals).

	Resilience
**Time by passive screen time**	
Time 2 by 1–3 h	9.41 (0.72–18.10)*
Time 2 by 3–5 h	9.60 (0.86–18.33)*
Time 2 by >5 h	6.80 (−2.22 to 15.83)
Time 3 by 1–3 h	−1.69 (−9.34 to 5.97)
Time 3 by 3–5 h	−4.35 (−12.21 to 3.51)
Time 3 by >5 h	−3.21 (−11.02 to 4.60)

The model accounts for baseline SDQ total score, four types of screen time, age, sex, and time; *p < 0.05.
